# Rare Case of Ulnar-Mammary-Like Syndrome With Left Ventricular Tachycardia and Lack of *TBX3* Mutation

**DOI:** 10.3389/fgene.2018.00209

**Published:** 2018-06-15

**Authors:** Anna Zlotina, Artem Kiselev, Alexey Sergushichev, Elena Parmon, Anna Kostareva

**Affiliations:** ^1^Almazov National Medical Research Centre, Saint Petersburg, Russia; ^2^ITMO University, Saint Petersburg, Russia; ^3^Department of Women’s and Children’s Health, Center for Molecular Medicine, Karolinska Institute, Solna, Sweden

**Keywords:** heart–hand syndromes, ulnar-mammary syndrome, *TBX3*, *SYNM*, intermediate filaments, ventricular tachycardia

## Abstract

“Heart–hand” type syndromes represent a group of rare congenital conditions that combine cardiac pathology (structural defect or arrhythmic disorder) and limb abnormality. Significant clinical variability and genetic heterogeneity typical for such syndromes complicate correct diagnosis, prognosis, and appropriate genetic counseling of the affected families. By now, only single genes have been unambiguously determined as a genetic cause of heart–hand syndromes and phenotypically similar conditions. In the present study, we report on a 25-year-old Russian female patient with a clinical picture resembling ulnar-mammary syndrome (UMS). Principal clinical manifestations included heart septal fibrosis and non-sustained left ventricular tachycardia combined with fifth finger camptodactyly, hypoplastic breast, abnormal teeth, and mental retardation. Target Sanger sequencing and array-based comparative genome hybridization confirmed the lack of pathogenic mutations and large-scale deletions in *TBX3* (12q24.21), the only gene known to be associated with UMS cases to date. Based on the results of whole-exome sequencing, 14 potential candidate variants were identified. Among them, a novel missense variant in *SYNM* gene (exon 1, c.173C > T, p.A58V), encoding intermediate filament protein synemin was characterized. Until the present, no association between *SYNM* mutations and congenital clinical syndromes has been reported. At the same time, taking into account synemin tissue-specific expression profiles and available data on abnormal knock-out mice phenotypes, we propose *SYNM* as a candidate gene contributing to the UMS-like phenotype. Further comprehensive functional studies are required to evaluate possible involvement of *SYNM* in genesis of complex heart-limb pathology.

## Introduction

Congenital heart disorders can represent isolated anomalies or be a part of complex syndromic phenotypes. “Heart–hand” syndromes (HHSs) are a group of rare congenital clinical conditions, where patients in addition to cardiac pathology (congenital heart defect and/or arrhythmic disorder) present with various abnormalities of limb skeleton, as well as additional dysmorphia (Holt and Oram, 1960; Ruiz de la Fuente and Prieto, 1980; Hollister and Hollister, 1981; Silengo et al., 1990; Šinkovec et al., 2005). Genetic basis of “heart–hand” type syndromes and phenotypically similar pathologies remains poorly understood with only a few causative genes or chromosomal loci identified. In particular, *TBX5* gene defects were shown to be responsible for the most common prototypical heart–hand syndrome type I, or Holt-Oram syndrome (HOS, MIM^1^ 142900) characterized by cardiac septal defects, conduction system disease and radial ray anomaly of forelimb skeleton (Basson et al., 1997; Li et al., 1997). *TBX5* belongs to an evolutionary conserved gene family encoding transcription factors with a DNA-binding domain, T-box, and plays various roles in developmental processes, including cardiogenesis and specification of forelimb identity (Rodriguez-Esteban et al., 1999; Bruneau et al., 2001). Truncation, missense, and splice site mutations in *TBX5* are well-described in HOS cases with a mutation type and position defining the severity of cardiac and skeletal phenotype (reviewed in Packham and Brook, 2003). Besides, the phenotype expression of Holt-Oram syndrome can be caused by interstitial chromosomal deletions in chromosomes 6 and 14 (Adamopoulos et al., 2004; Le Meur et al., 2005).

Mutations in another transcription factor gene – *TFAP2B –* expressed in neural crest cells were shown to be responsible for a phenotypically distinct autosomal dominant disorder, Char syndrome (MIM 169100) characterized by patent ductus arteriosus, fifth digit middle phalangeal hypoplasia, and additional facial dysmorphism (Char, 1978; Satoda et al., 2000). By contrast, Slovenian type of heart–hand syndrome (HHS IV, MIM 610140) combining conduction system disease, atrial and ventricular tachyarrhythmias, dilated cardiomyopathy, and brachydactyly proved to be a laminopathy caused by particular mutations in *LMNA* gene encoding a structural intermediate filament (IF) protein of a nuclear lamina (Šinkovec et al., 2005; Renou et al., 2008; Zaragoza et al., 2017).

Ulnar-mammary syndrome (UMS, also referred to as Schinzel syndrome or Pallister UMS, MIM 181450) represents a similar autosomal dominant condition also involving hands and the heart. Key UMS clinical features usually include ulnar ray defects (ranging from fifth finger deformities to complete absence of the ulna), hypoplasia of mammary and apocrine glands, abnormal teeth, genital hypoplasia, and puberty delay. At the same time, additional manifestations comprise myocardial pathology, namely cardiac conduction abnormality and/or congenital heart defect (Meneghini et al., 2006; Linden et al., 2009). The syndrome is associated with mutations in *TBX3* gene (locus 12q23-24.1), another member of T-box gene family (Bamshad et al., 1997, 1999). Indeed, a particular vital role of *TBX3* was shown for limb and mammary gland development, differentiation of cardiac conduction system and heart looping and growth (reviewed in Washkowitz et al., 2012). In addition to single nucleotide mutations, there are several reported cases of contiguous microdeletions at 12q24.21 locus encompassing both *TBX3* and *TBX5* genes and giving rise to phenotypes that combine UMS and Holt-Oram syndrome features (Borozdin et al., 2006; Alby et al., 2013; Bogarapu et al., 2014; Iwanicka-Pronicka et al., 2016).

Numerous phenotypically overlapping clinical cases were reported with undetermined genetic basis (Ruiz de la Fuente and Prieto, 1980; Hollister and Hollister, 1981; Silengo et al., 1990; Morava et al., 2003; Demura et al., 2010; Nanda et al., 2010). Identification of new deleterious genetic variants, candidate genes and modifiers, which became possible due to high-throughput sequencing approaches and array-based comparative genome hybridization (array-CGH), proves to be helpful for meeting the diagnostic challenge and enables new insights into molecular and cellular mechanisms underlying combined limb-heart malformations (Liu et al., 2017; Zaragoza et al., 2017).

In the present study, we report on a 25-year-old woman with a clinical picture resembling UMS. Main clinical manifestations included pathology of 5th digits, hypoplasia of the mammary glands, mental retardation, and heart septal fibrosis combined with non-sustained ventricular tachycardia. Sanger sequencing and array-CGH allowed to exclude causative role of *TBX3*. Based on the results of whole-exome sequencing (WES), we describe a novel missense variant in *SYNM* gene encoding IF protein synemin and discuss its potential involvement in the patient’s phenotype.

## Materials and Methods

Standard karyotyping was carried out on GTG-banded metaphase chromosomes obtained from phytohemagglutinin-stimulated peripheral blood lymphocytes. Oligonucleotide array-based CGH was performed using Agilent 8x60K array platform with median probe spacing 41 kb (SurePrint G3 Human CGH Microarray, Agilent Technologies, Santa Clara, CA, United States). The data obtained was processed and analyzed using CytoGenomics Software (v3.0.1.1, Agilent Technologies). Copy number variations (CNVs) were called using an aberration detection statistical algorithm ADM-2, with a sensitivity threshold of 6.0.

Bidirectional Sanger sequencing was applied to search for single-nucleotide genetic variants in *TBX3* gene (Gene ID: 6926, NG_008315.1^2^) using BigDye Terminator Sequencing Kit (Applied Biosystems) and Genetic Analyzer AB3100 (Applied Biosystems/Hitachi, Japan). WES DNA-library was prepared using the SureSelect^XT^ Human All Exon v6 r2 (60 Mbp) target enrichment kit (Agilent Technologies, Santa Clara, CA, United States). Sequencing run was carried out with SBSv4 chemistry and the Illumina HiSeq instrument (Illumina, San Diego, CA, United States). Alignment was performed using Burrows-Wheeler Aligner (BWA-MEM-0.7.1, Li and Durbin, 2009) with GRCh37/hg19 human genome assembly as a reference after that the data processing (Picard 2.8.3) and variant calling (GATK 3.7.) was performed according to Broad institute GATK Best Practice. Variant annotation was done using Annovar (Wang et al., 2010). Variant population frequencies were evaluated based on ExAc^3^ and gnomAD^4^ resources, functional prediction was made based on dbNSFP (v3.3a). Data on tissue-specific gene expression profiles were taken from UniProt^5^, GNF gene expression atlas, Human Protein Atlas^6^. Additionally, variants were evaluated based on expression rank in heart tissue according to GTEx dataset. Average target region coverage was ∼ x150 with 95% of the target region being covered to a depth of 20 or more.

Written informed consent was obtained from the patient for the genetic study and publication of images. The study was performed according to Helsinki Declaration and study approval was obtained from Institutional Ethical Review Board at the Almazov National Medical Research Centre in St. Petersburg.

## Results

### Clinical Case

**FIGURE 1:**
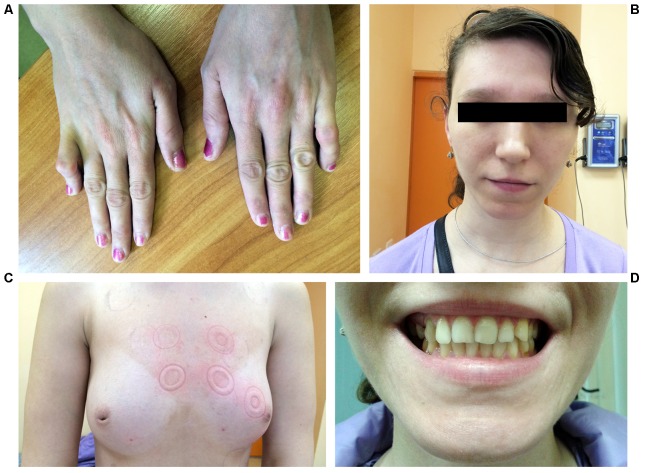
Photographs representing phenotype of the patient. **(A)** Hands demonstrating fifth finger camptodactyly. **(B)** Facial features include wide-set eyes, strabismus, a broad nasal tip, and thin upper lip vermilion. **(C)** Hypoplastic breast with inverted nipples. **(D)** Dental abnormalities involving canines and back teeth.

A 25-year-old woman was hospitalized due to frequent premature ventricular beats of high grade (17,000 per day) and repeated episodes of bidirectional non-sustained ventricular tachycardia without syncope. Echocardiography revealed enlarged left ventricular dimension and local ventricular wall thinning. Upon routine clinical examination bilateral symmetrical hand abnormality was noted, namely the fifth finger camptodactyly (**Figure 1A**). Additionally, hypoplasia of the breast with inverted nipples was observed (**Figure 1C**). Facial features included wide-set eyes, a broad nasal tip and thin upper lip vermilion and strabismus (**Figure 1B**). Dental abnormalities were represented by tooth malalignment and hypoplasia involving canines and back teeth (**Figures 1C,D**). No defects were documented in her lower limbs. Apart from physical defects, intellectual deficit was noted and included mild mental retardation and learning disabilities. Family history reported that proband’s mother died due to congenital heart defect and congestive heart failure at the age of 30. Grandmother from mother side was not affected. No other relatives were available for examination. Due to the lack of family data, it is hard to conclude the mode of inheritance unambiguously. However, keeping in mind the mother’s phenotype, the dominant inheritance could be suggested (**Supplementary Figure S1**).

### Genetic Studies

Standard cytogenetic analysis showed normal female karyotype. Taking into consideration the patient’s clinical phenotype similar to UMS, the next step was to screen the *TBX3* locus for genome variations. However, bidirectional Sanger sequencing of *TBX3* protein-coding regions including 3′- and 5′- flanking intronic sequences did not reveal any known pathogenic mutations or variants of uncertain significance. High-resolution microarray-CGH analysis allowed to exclude a whole-gene *TBX3* deletion as well as other causative microimbalances over ∼100–150 kb in size. These findings imply that the patient’s syndromic phenotype is unlikely to be caused by *TBX3* deficiency and is rather due to another genetic defect.

**FIGURE 2:**
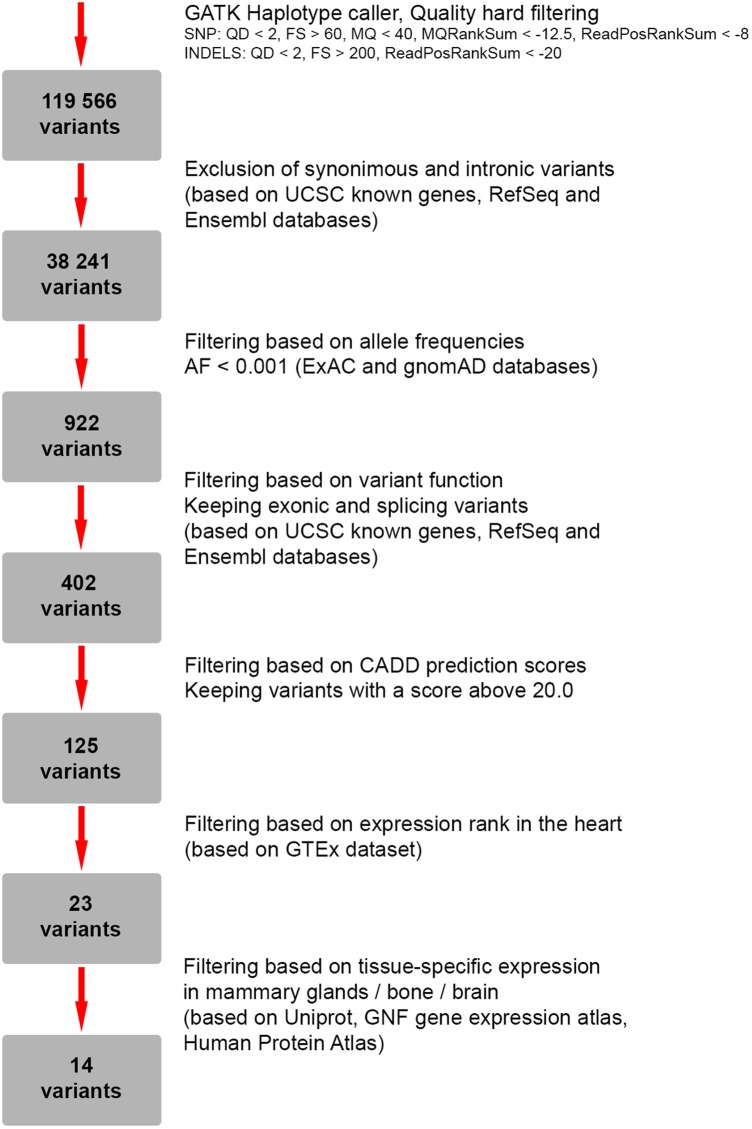
Flowchart representing a strategy for filtering of genetic variants identified by whole-exome sequencing (WES).

To search for candidate genes, WES was performed. The detailed workflow of filtering strategy with total numbers of variants left after each step is depicted as a flowchart (**Figure 2**). The called variants were filtered according to their exonic function and population frequencies so that deep intronic variants, exonic synonymous substitutions and all variants with allele frequency 0.1% and higher were excluded from the further analysis. As a result, rare protein-changing variants (missense, frameshifts, nonsense, and predicted splice sites) were further evaluated based on gene functions, clinical annotations, mode of inheritance and prediction of variant functional effect (**Supplementary Table S1**). We did not identify any genes responsible for “hear-hand” syndromes or syndromic conditions with overlapping cardiopathology, limb skeletal manifestation or malformed breast, including *TBX5* (Holt-Oram syndrome), *LMNA* (Slovenian type of heart–hand syndrome), *TFAP2B* (Char syndrome), *TP63* [Limb-mammary syndrome (MIM 603543), ADULT syndrome (MIM 103285)].

**FIGURE 3:**
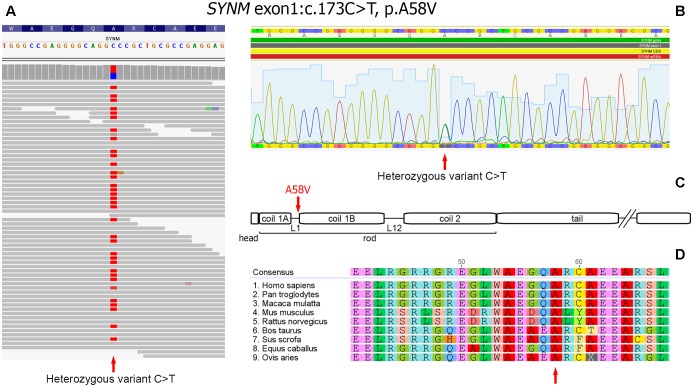
Heterozygous missence variant in *SYNM* c.173C > T (p.A58V). **(A)** Identification of the variant by WES. The variant position is pointed by an arrow. **(B)** Validation of WES results by Sanger sequencing: a fragment of sequencing chromatogram. Heterozygous C/T variant is pointed by an arrow. **(C)** Position of the amino acid change in synemin protein (shown by an arrow). Protein domain structure is depicted according to UniProt (http://www.uniprot.org). **(D)** Evolutionary conservation analysis of the amino-acid position across mammalian species (shown by an arrow).

As a next step we focused on the genes highly expressed in tissues/organs affected and known to be involved in their morphogenesis, pathogenesis and functioning. After the filtering process, 14 candidate variants were selected (**Supplementary Table S2**). Among them, a heterozygous missense variant in *SYNM* gene (exon 1, c.173C > T, p.A58V), encoding IF protein synemin was selected as a favorable candidate and validated by Sanger sequencing (**Figures 3A,B**). In contrast to other IF proteins that tend to be tissue-specific, synemin was detected in a broad spectrum of tissues and organs including heart, breast, adipose tissue, bone cells, and brain. Though no clinical mutations in *SYNM* have been reported by now, the data on severe cardiac and osteopenic phenotypes in *SYNM* knockouts have been accumulated (for details, see section “Discussion”).

Synemin presents all functional domains typical for IFs (Bellin et al., 1999; Mizuno et al., 2001; Titeux et al., 2001) and the identified genetic variant is mapped to the rod domain, on the border of a short polypeptide linker L1 and α-helical segment 1B (**Figure 3C**). The variant is currently absent in publicly available databases of normal or clinical SNPs such as Clinvar, dbSNP, ExAC, 1000 Genomes. Alignment of synemin protein sequences demonstrated evolutionary conservation at this amino acid position across mammalian species (**Figure 3D**). According to SIFT, FATHMM, MetaLR and M-CAP functional prediction tools, the variant is evaluated as deleterious. In dbSNP database, we found a rare unclassified missence variant at the same nucleotide position of *SYNM* (rs1367107502, MAF: 0.00002, TOPMED project), where, however, another allele (C > A) and residue (p.A58D) change took place. Based on ACMG guideline for the interpretation of sequence variants (Richards et al., 2015), the variant described here should be classified as a variant with unknown significance (VOUS) and needs to be further functionally tested using appropriate animal or cell culture models.

## Discussion

Ulnar-mammary syndrome represents a rare congenital multi-systemic disorder characterized by incomplete penetrance and significant intra- and inter-familial clinical variability (Bamshad et al., 1999; Wollnik et al., 2002; Linden et al., 2009), which complicates accurate diagnosis and appropriate genetic counseling. The presence of limb abnormalities, especially involving the digits of the hand’s ulnar ray, and mammary gland hypoplasia implies the consideration of UMS. In the present study we describe a female patient with UMS-like phenotype manifestations including abnormalities of hand fifth digits, breast, teeth, and cardiac phenotype. The latter in form of septal fibrosis and non-sustained ventricular tachyarrhythmia is consistent with UMS phenotype, though heart pathology is rather rare component of the syndrome. In particular, two confirmed cases of a ventricular septal defect and one case of conduction abnormality in the form of Wolff–Parkinson–White syndrome were previously reported in UMS pediatric patients (Meneghini et al., 2006; Linden et al., 2009). Some clinical manifestations of our patient such as mental retardation and strabismus are not typically described as a part of UMS syndrome. To the best of our knowledge, the only UMS case combined with mental retardation was not caused by a *TBX3* point mutation, but by a contiguous 1.28 Mb deletion at 12q24.2 chromosomal region (Klopocki et al., 2006). With regard to eye anomalies, congenital unilateral anophthalmia of unknown etiology was also noted once in a UMS patient (Linden et al., 2009).

To date, *TBX3* is the only gene associated with UMS disorder. More than 20 *TBX3* pathogenic mutations or large-scale gene aberrations have been reported in association with UMS cases with haploinsufficiency being regarded as a main disease-causing mechanism (Bamshad et al., 1999; Wollnik et al., 2002; Klopocki et al., 2006; Linden et al., 2009; Alby et al., 2013; Tanteles et al., 2017). At the same time, in our patient the lack of *TBX3* mutation was confirmed by target Sanger sequencing, WES and CGH-microarray results. Notably, single cases of unclassified clinical conditions phenotypically similar to UMS have been earlier reported, where a *TBX3* defect was not identified as a genetic cause (Morava et al., 2003). Therefore, such conditions might be regarded as an UMS-like disorder with apparently another genetic basis, possibly linked to other transcription factors or structural genes mutations.

In present study no morbid or unclassified variants were revealed in the genes underlying common heart–hand syndromes. Given the several criteria for variant evaluation such as absence of population frequency and prediction of functional effects, as well as protein tissue-specific expression profiles and literature data, we propose *SYNM*, encoding IF synemin, as a potentially novel candidate gene contributing to UMS-like condition. High level of synemin expression is shown in various types of tissues including those that are involved in the patient’s syndromic phenotype. In particular, initially synemin was described as an IF protein abundant in all muscle cells where in case of striated muscles it predominantly localized in the region of Z-disk, costamers and intercellular junctions, including intercalated disks of cardiomyocytes (Granger and Lazarides, 1980; Bilak et al., 1998; Bellin et al., 2001; Hirako et al., 2003). Since then, a list of synemin-positive cell types has been significantly extended and included mammary glands, adipose tissue, osteoblasts, some neural cells (The Human Protein Atlas; Hirako et al., 2003; Moorer et al., 2016; reviewed in Paul and Skalli, 2016).

Despite the absence of so far reported cases of *SYNM* mutations and their clinical phenotype, there are comprehensive data on abnormalities in synemin knock-out mice (Li et al., 2014; García-Pelagio et al., 2015, 2018; Moorer et al., 2016). Taking into consideration the prominent cardiac phenotype of our patient, it is of importance to note that the mice lacking synemin (synm-/-) demonstrate structural and functional abnormalities in the heart and myopathic changes (García-Pelagio et al., 2015, 2018). Absence of synemin in mice causes left ventricular remodeling, contractile and systolic dysfunction at 3 and 12–16 months of age with subsequent left ventricular hypertrophy and dilatation (García-Pelagio et al., 2018). *In vitro* examination of cardiomyocytes isolated from such knock-outs demonstrated the decreased calcium transients and contractility. Finally, synemin-null heart was characterized by alterations in a level of some signaling molecules (PKA-RII, ERK, and p70S6K) vital for cardiomyocyte function, which conforms to the previous knowledge on A-kinase anchoring properties of synemin in the heart (Russell et al., 2006). Depending on tissue origin, being unable to self-assemble into filaments, synemin co-polymerizes with other IF representatives, namely with desmin and vimentin (Bellin et al., 1999; Titeux et al., 2001). Thus, some other skeletal and cardiac muscle pathologies such as desmin-related myopathies are accompanied by altered synemin expression and cell distribution (Carlsson et al., 2002; Olivé et al., 2003).

In light of skeleton pathology, recent comprehensive study of synemin-null mice model demonstrated a prominent role of synemin in bone physiology (Moorer et al., 2016). That is, the animals were shown to suffer from osteopenia due to a substantial reduction in trabecular bone mass accompanied by the reduced osteoblast activity and number *in vivo* (Moorer et al., 2016). *Ex vivo* experiments on the isolated primary osteoblasts confirmed impaired proliferation of synemin -/- cells as compared to the wild-type but, surprisingly, revealed their elevated osteogenic differentiation capacity, thus, pointing to synemin involvement in osteoblast differentiation (Moorer et al., 2016). Similar results implying a role of synemin in a cell self-maintenance/differentiation balance were obtained for muscle satellite cells (Li et al., 2014) and glioblastoma cells (Pitre et al., 2012).

Accumulated data on synemin expression profiles, interacting partners and knock-out phenotypes demonstrate both structural and signaling roles of synemin during development or in adult tissues, including the role in structure and function of cardiac muscle and bone formation. Until the present, co-occurrence of synemin mutations with congenital clinical syndromes such as combined heart-limb pathology has not been yet reported. At the same time, the involvement of IF proteins in heart–hand syndrome phenotypes has already been shown. In particular, two specific mutations in *LMNA* gene encoding a nuclear lamina protein were identified as a genetic cause of the HHS type IV characterized by tachyarrhythmia, cardiomyopathy, and brachydactyly (Renou et al., 2008; Zaragoza et al., 2017). The findings obtained in the present study support a possible association between an IF gene and combined heart–hand malformation. The potential molecular mechanism underlying this association could involve cell proliferation/differentiation process in progenitor cells during development similar to that reported for *LMNA* mutations responsible for different laminopathy conditions (Malashicheva et al., 2015).

## Concluding Remarks

Here, we report on a non-typical case of UMS with prominent cardiac manifestation and mild mental retardation. In contrast to common UMS cases, the described phenotype is not associated with *TBX3* mutation or large-scale deletion. The results of WES data analysis and a review of literature data pointed to an IF gene, *SYNM*, as a potential new candidate gene contributing to the UMS-like condition. To prove a possible association between *SYNM* mutation and UMS-like condition further functional cell studies are planned.

## Data Availability

The dataset of analyzed whole-exome sequencing results is included in the manuscript and the supplementary files. The raw data supporting the conclusions of this manuscript will be made available by the authors, without undue reservation, to any qualified researcher.

## Author Contributions

EP and AaK patient workup and genetic counseling. AZ and AmK genetic analysis. AmK, AS, AZ, and AaK NGS data processing and analysis. AZ and AaK design of the study. AaK coordination of the study. AZ drafted the manuscript. AZ, AmK, AS, EP, and AaK final revision and approval of the manuscript. All authors agreed to be accountable for all aspects of the work.

## Conflict of Interest Statement

The authors declare that the research was conducted in the absence of any commercial or financial relationships that could be construed as a potential conflict of interest.

## Footnotes

1 Online Mendelian Inheritance (OMIM) available at: https://omim.org/

2 https://www.ncbi.nlm.nih.gov/gene/6926

3 Exome Aggregation Consortium available at: http://exac.broadinstitute.org/

4 Genome Aggregation Database http://gnomad.broadinstitute.org/

5 The Universal Protein Resource available at: http://www.uniprot.org

6 The Human Protein Atlas available at: https://www.proteinatlas.org/

## Supplementary Material

FIGURE S1 Pedigree chart of the reported family. Gray color indicates mother’s phenotype partially overlapping with the proband’s clinical picture. Question mark indicates unavailable data on the phenotype.

TABLE S1 Whole-exome sequencing data: a list of genetic variants filtered according to their exonic function and population allele frequencies. As a result, 402 rare protein-changing variants (missense, frameshifts, nonsense and predicted splice sites) with AF < 0.001 (ExAc and gnomAD databases) were selected.

TABLE S2 Whole-exome sequencing data: a final list of 14 candidate genetic variants filtered based on CADD prediction scores, expression rank in the heart and tissue-specific expression in mammary glands, bone and brain (GTEx dataset, Uniprot, GNF gene expression atlas, Human Protein Atals).
