# Supplementary figures and images for: Rare Case of Ulnar-Mammary-Like Syndrome With Left Ventricular Tachycardia and Lack of TBX3 Mutation
Source: Front Genet. 2018 Jun 15;9:209. doi: 10.3389/fgene.2018.00209 (PMC6013977; doi:10.3389/fgene.2018.00209)

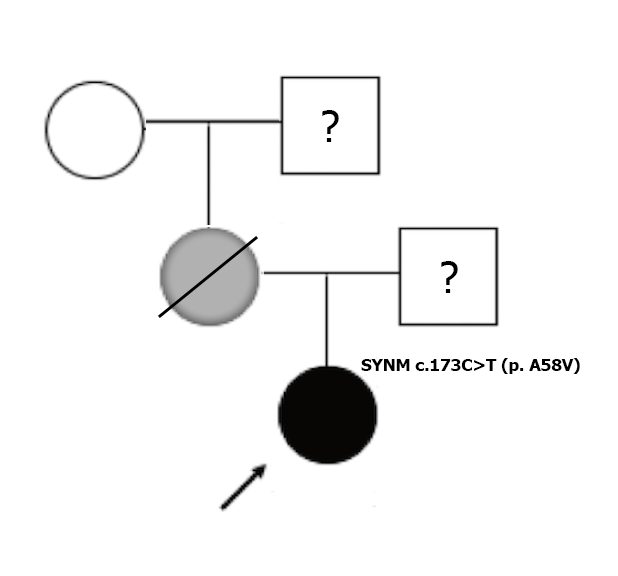

Supplement: FIGURE S1 — Pedigree chart of the reported family. Gray color indicates mother’s phenotype partially overlapping with the proband’s clinical picture. Question mark indicates unavailable data on the phenotype. [file Image_1.TIF]
