# Supplementary material for: Rare Case of Ulnar-Mammary-Like Syndrome With Left Ventricular Tachycardia and Lack of TBX3 Mutation
Source: Front Genet. 2018 Jun 15;9:209. doi: 10.3389/fgene.2018.00209 (PMC6013977; doi:10.3389/fgene.2018.00209)
Supplement: Supplementary file 4 [file Data_Sheet_1.DOCX]

***Supplementary Material***

**Rare case of ulnar-mammary-like syndrome with left ventricular tachycardia and lack of *TBX3* mutation**

**Anna Zlotina^*^, Artem Kiselev, Alexey Sergushichev, Elena Parmon, Anna Kostareva^*^**

*** Correspondence:**

Dr. Anna Zlotina

anna-zlotina@yandex.ru

Dr. Anna Kostareva

akostareva@hotmail.com


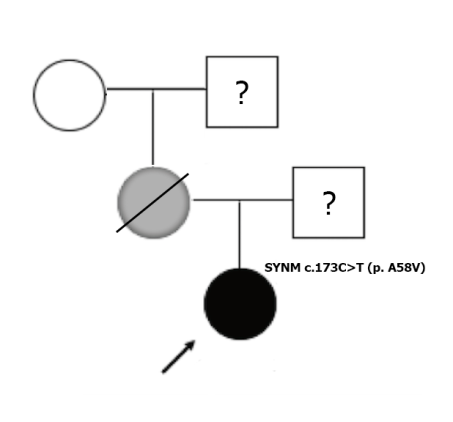


**Supplementary Figure 1.** Pedigree chart of the reported family. Grey color indicates mother’s phenotype partially overlapping with the proband’s clinical picture. Question mark indicates unavailable data on the phenotype.
